# Immunoglobulin Free Light Chains and GAGs Mediate Multiple Myeloma Extracellular Vesicles Uptake and Secondary NfκB Nuclear Translocation

**DOI:** 10.3389/fimmu.2014.00517

**Published:** 2014-10-27

**Authors:** Giuseppe Di Noto, Marco Chiarini, Lucia Paolini, Elena Laura Mazzoldi, Viviana Giustini, Annalisa Radeghieri, Luigi Caimi, Doris Ricotta

**Affiliations:** ^1^Department of Molecular and Translational Medicine, Faculty of Medicine, University of Brescia, Brescia, Italy; ^2^CREA, Diagnostic Department, Azienda Ospedaliera Spedali Civili di Brescia, Brescia, Italy

**Keywords:** extracellular vesicles, serum-free light chain, glycosaminoglicans, NfκB, multiple myeloma

## Abstract

Multiple myeloma (MM) is a hematological malignancy caused by a microenviromentally aided persistence of plasma cells in the bone marrow. Monoclonal plasma cells often secrete high amounts of immunoglobulin free light chains (FLCs) that could induce tissue damage. Recently, we showed that FLCs are internalized in endothelial and myocardial cell lines and secreted in extracellular vesicles (EVs). MM serum derived EVs presented phenotypic differences if compared with monoclonal gammopathy of undetermined significance (MGUS) serum derived EVs suggesting their involvement in MM pathogenesis or progression. To investigate the effect of circulating EVs on endothelial and myocardial cells, we purified MM and MGUS serum derived EVs with differential ultracentrifugation protocols and tested their biological activity. We found that MM and MGUS EVs induced different proliferation and internalization rates in endothelial and myocardial cells, thus we tried to find specific targets in MM EVs docking and processing. Pre-treatment of EVs with anti-FLCs antibodies or heparin blocked the MM EVs uptake, highlighting that FLCs and glycosaminoglycans are involved. Indeed, only MM EVs exposure induced a strong nuclear factor kappa B nuclear translocation that was completely abolished after anti-FLCs antibodies and heparin pre-treatment. The protein tyrosine kinase c-src is present on MM circulating EVs and redistributes to the cell plasma membrane after MM EVs exposure. The anti-FLCs antibodies and heparin pre-treatments were able to block the intracellular re-distribution of the c-src kinase and the subsequent c-src kinase containing EVs production. Our results open new insights in EVs cellular biology and in MM therapeutic and diagnostic approaches.

## Introduction

Extracellular vesicles (EVs) are emerging as pleiotropic actors in intercellular signaling. The discovery of exosome (a specific subpopulation of EVs) mediated transfer of specific mRNAs and miRNAs (1) opened the door for EVs research in cancer biogenesis and progression. Indeed, EVs carry a broad number of cargos such as molecules that take part in determining cell identity, signaling pathways, and deregulation in many cancer forms.

Exosomes are the most attractive models in EVs interplay studies because they originate from specific intracellular compartments such as endosomes and multivesicular bodies. Moreover, growing evidences show that tumors constitutively shed exosomes with immunosuppressive effects regulating tumor growth, invasion, angiogenesis, and metastasis (2).

Evs involvement in multiple myeloma (MM) is starting to be unveiled too. It has been published that normal and MM bone marrow–mesenchymal stem cells (BM–MSC)-derived exosomes differentially affect MM cell homing and growth *in vivo*. A novel mechanism by which BM–MSCs play an oncogenic role in MM is the epigenetic transfer of exosomal miRNA to the tumor clone (3).

Label-free relative quantitative proteomic analysis of circulating MM EVs identified an EVs specific protein content (4), while we showed that monoclonal gammopathy of undetermined significance (MGUS) derived EVs are different from those produced in MM (5). Indeed, we observed a significant increase in EVs production in MM patients, and monoclonal immunoglobulin free light chains (FLCs) were rerouted in the extracellular space via EVs. The serum vesicles from MM patients contained the “proto-oncogene” c-src kinase compared to MGUS and control subjects. It has been shown that constitutive activation of c-src promotes cell survival, proliferation, and chemoresistance in MM (6, 7) and our finding highlights the involvement of EVs as c-src kinase transactivating carriers (5). Specific targeting of EVs is probably related to their cellular origin and function. Cell-specific proteins represent a means by which exosomes can specifically dock various recipient cells by either interaction with cell-surface adhesion molecules or through interaction with cell-surface heparan sulfate proteoglycans (2). Phagocytosis is also involved in specific EVs uptake into the cell in a cell-type dependent manner (8). As potential targeting sites, heparan sulfate proteoglycans have been shown to function as internalizing receptors of cancer cell-derived EVs (9). Furthermore, glycosaminoglycans (GAGs) are involved in FLCs fibrillation and amyloidogenesis: the evidences that amyloid-related light chain proteins and GAGs, particularly heparin, interact, suggest that the therapeutic use of GAGs antagonists to prevent amyloidosis (10).

Shed syndecan-1 (CD138), a GAG binding protein, is known to actively promote myeloma tumor growth, angiogenesis, and metastasis in MM (11) and patients with high-serum levels of heparanase develop more severe illness. The mechanism of heparanase aggressive phenotype priming is in part due to synergy with the heparan sulfate proteoglycan syndecan-1 to create a niche within the bone marrow microenvironment, further driving myeloma growth and dissemination (12, 13). The evidences that syndecan-1 and heparanase are also involved in exosomes generation/uptake thus indicate a potential mechanism in MM EVs trafficking (14).

In the present work, we studied the biological activity of “as pure as possible” serum EVs. As pure as possible, thus, named EVs instead of exosomes, because purified serum samples may contain protein aggregates and ectosomes. At the moment, separation of ectosomes and exosomes with the same size and density is still difficult. To investigate the EVs effect on endothelial and myocardial cells we used gradient fraction grade purity EVs populations and found that MM EVs induce a significant higher proliferation rate than MGUS EVs. We also found that while MM EVs are internalized, MGUS EVs are not. Internalization was blocked with anti-FLCs antibodies or heparin pre-treatment. More strikingly, we found that MM EVs induced a c-src kinase containing EVs release and nuclear factor kappa B (NfκB) nuclear translocation in cultured cells and both processes were blocked by anti-FLCs antibodies and heparin treatment.

In summary, this paper reports a novel potential use of anti-FLCs antibodies in MM patients together with heparin analogs. Furthermore, we propose the EVs-NfκB translocation assay for monitoring MGUS patients.

## Materials and Methods

### Ethics statement and sample collection

Serum samples were collected in the Laboratory of Clinical Biochemistry, Azienda Ospedaliera Spedali Civili of Brescia (AOSCB). After routine analysis, waste serum samples were coded, anonymized and frozen at −80°C: monoclonal components were detected by high-resolution agarose gel electrophoresis/immunofixation (Interlab). Immunoglobulin FLC concentration was measured by particle enhanced nephelometry (Freelite assay, The Binding Site) on a Behring BNII Nephelometer (Dade Behring). The institutional review board of Azienda Ospedaliera Spedali Civili of Brescia approved the study in adherence with the Declaration of Helsinki (REC number: SFLC01). All traceable identifiers were removed before analysis to protect patient confidentiality and all samples were analyzed anonymously. Samples analyzed include six patients with diagnosed MM (MM1-6), three MGUS patients (MGUS1–3), and three healthy donors used as controls (c1–3).

### EVs purification and fractionation

To obtain heterogenous EVs populations, 1 ml serum sample was processed with serial centrifugation steps (800 × *g* for 30 min, 16,000 × *g* for 45 min, 100,000 × *g* for 2 h) and the pellets were re-suspended in 50 μl PBS 1× supplemented with 1:1000 Protease Inhibitor Cocktail (P.I., Sigma). Reducing sample buffer was added and the samples were boiled 5 min at 95°C. Samples were electrophoresed in SDS-PAGE and analyzed by western blot (WB). The heterogeneous EVs populations were subsequently processed for further fractionation using a discontinuous sucrose gradient as described in the next paragraph. Samples were normalized for protein content (Bradford assay) whenever possible; in alternative, equal volumes of each sample were loaded on an acrylamide–bisacrylamide gel.

### Sucrose gradient

The heterogeneous EVs populations (200 μg of pelleted proteins) were re-suspended in 800 μl buffer A (10 mM Tris–HCl 250 mM sucrose, pH 7.4), loaded at the top of a discontinuous sucrose gradient (15, 20, 25, 30, 40, 60% sucrose in 10 mM Tris–HCl, pH 7.4) and centrifuged at 100,000 × *g* for 16 h at 4°C (rotor MLS 50, Beckman Optima MAX). Twelve fractions with equal volumes (400 μl) were collected from the top of the gradient, and the vesicles were pelleted by ultracentrifugation (100,000 × *g* for 2 h). The pellets were re-suspended in 50 μl of 100 mM Tris, 150 mM NaCl, 1 mM EDTA supplemented with 1:1000 Protease Inhibitor Cocktail (P.I., Sigma). Reducing sample buffer was added and the samples were boiled 5 min at 95°C. Samples were electrophoresed in SDS-PAGE and analyzed by WB. Positive fractions containing EV markers (from 6 to 9, 1.11–1.22 g/cm^3^) were further investigated by atomic force microscopy (AFM), scanning electron microscopy (SEM), and lipid fluorescent labeling.

### Scanning electron microscopy (SEM)

Extracellular vesicles were purified from 1 ml serum with serial centrifugations and fractionated onto a discontinuous sucrose gradient as described before. Fractions known to be positive for EV markers were ultra-centrifuged (100,000 × *g* for 2 h), pellets were re-suspended in 200 μl PBS 1×, and centrifuged (400 × *g* for 5 min) with a Cytospin4 centrifuge (The Thermo Scientific). Samples were fixed with 2.5% glutaraldehyde (Sigma) in PBS 1× for 1 h. After washing twice with PBS 1×, the fixed samples were dehydrated with an ascending sequence of ethanol (25, 50, 75, 90, 100%). Ethanol was then washed away with high-pressure liquid carbon dioxide (critical point dryer CO_2_, Balzers Union). Samples were analyzed by SEM after gold sputtering (Balzers Union Sputtering System SCD 040), using a Philips 501 SEM operating at 15 kV.

### Atomic force microscopy (AFM)

Extracellular vesicles were re-suspended in 50 μl of 100 mM Tris, 150 mM NaCl, 1 mM EDTA, and diluted 1:10 with deionized water. Five to 10 μl of samples were then spotted onto freshly cleaved mica sheets (Grade V-1, thickness 0.15 mm, size 15 mm × 15 mm). All mica substrates were dried at room temperature and analyzed using a JEOL JSPM-5200, using a Veeco AFM tip or a MikroMasch AFM tip. Images were snapped in tapping mode, scan size ranged from 0.3 to 15 μm and scan speed ranged from 0.6 to 3.3 ms × clock.

### Fluorescent labeling

Extracellular vesicles were re-suspended in Diluent C (PKH67 Green Fluorescent cell linker, Sigma) to a 70 μl final volume. 1.7 μl of PKH67 green fluorescent dye was added to each sample and incubated at room temperature for 10 min. In alternative, we labeled EVs with PKH26 red fluorescent dye. The reaction was stopped adding 70 μl of 1% BSA in PBS 1×. EVs were centrifuged at 100,000 × *g* for 2 h.

### Flow cytometry

#### EVs analysis

Forty microliters of Exo-Flow FACS Magnetic beads [9.1 μm, 400 μl at 10 mg/ml, 1.6 × 10^7^ beads/ml (SBI, System Bioscience)] were coupled with 10 μl of anti-CD63 biotinylated antibody following manufacturer instructions. Afterwards, 100 μg (protein concentration) of EVs were incubated on a rotating rack at 4°C overnight for CD63 positive EVs capture. Exosomes-coated beads were stained on ice for 2 h with PKH26 (Sigma, 1 μl/80 μg of EVs proteins) and with 10 μl of Exo-FITC exosome stain (SBI, System Bio-Science) and then analyzed on a FACSCanto II flow cytometer (BD Biosciences) using the FACSDiva software 2.56 (BD Biosciences).

#### Re-suspended cells analysis

Human vein endothelial cells (HVEC) were incubated with PKH67-labeled EVs for 4 h at 37°C; subsequently, cell monolayers were washed with PBS 1×, detached using trypsin, and re-suspended in PBS 1×. Flow-cytometry analysis was performed on a FACSCanto II (BD Biosciences) using the FACSDiva software 2.56 (BD Biosciences). Gate was set on living cells based on forward/side scatter properties and a minimum of 10^3^ events within the gated live population were collected per sample. The intracellular PKH67-labeled EVs were measured by the peak fluorescence intensity shift of PKH67, calculated by the geometric mean of the population. HVECs were stained with mouse anti CD31-APC (allophycocyanin) primary antibody. Internalized EVs were stained with or without PKH67-labeled EVs (unlabeled EVs were used as cell auto-fluorescence control).

### EVs acetylcholinesterase activity evaluation

Extracellular vesicles acetylcholinesterase activity was assayed following a previously described procedure (15, 16). Briefly, EVs pellets from 1 ml serum were re-suspended in 100 μl of PBS 1× and incubated with 1.25 mM acetylthiocholine and 0.1 mM 5,5-dithiobis (2-nitrobenzoic acid) in a final volume of 400 μl. The incubation was carried out in cuvettes at 37°C and the absorbance change was monitored every 5 min at 412 nm (0–30 min). The data represent the enzymatic activity after 20 min.

### Protein/lipid ratio calculation

Extracellular vesicles protein concentration from controls (three patients), MGUS (three patients), and MM (six patients) serum was quantified with Bradford assay. Samples were normalized for protein content (200 μg). EVs were PKH67-labeled as described and re-suspended in 20 μl of loading dye [36% Tris–acetate–EDTA (TAE), 14% H_2_O, 50% glycerol]. Ten microliters of fluorescent EVs were spotted on a nitrocellulose membrane and dots fluorescent signal were acquired using a G:Box Chemi XT Imaging system (Syngene). Signals were quantified with the Gene Tools program. Protein content/lipid fluorescent signal ratio was calculated.

### Cell culture

H9C2 (ATCC CRL-1446; tissue: heart/myocardium; cell type: myoblast) cells were grown in Dulbecco’s modified eagle’s medium (DMEM) supplemented with 10% fetal bovine serum (FBS) (Lonza), 1% penicillin/streptomycin (Lonza), 1% glutamine (Lonza); HVECs (17) were grown in RPMI 1640 supplemented as DMEM, at 37°C, 5% CO_2_.

### Fractionation of cytoplasmic, membranous, and nuclear components

HVEC monolayer were washed two times with cold PBS 1×, scraped with PBS-0.1 mM EDTA, and ultra-centrifuged at 800 × *g* for 10 min at 4°C. The pellet was re-suspended in harvest buffer (10 mM Hepes pH 7.9, 50 mM NaCl, 0.1 mM EDTA, 0.5% Triton X-100, and freshly added 1 mM DTT, 10 mM tetrasodium pyrophosphate, 10 mM NaF), incubated on ice for 5 min, and centrifuged at 800 × *g* for 10 min at 4°C. The supernatant contains cytosolic and membranous proteins while the pellet, containing nuclei, was washed with Buffer A (10 mM HEPES pH 7.9, 10 mM KCl, 0.1 mM EDTA, 0.1 mM EGTA, and freshly added 1 mM DTT, 1 mM PMSF, 4 μg/ml aprotinin, 2 μg/ml pepstatin) and centrifuged at 800 × *g* for 10 min at 4°C. After centrifugation, the pellet was re-suspended in buffer C (10 mM HEPES pH 7.9, 500 mM NaCl, 0.1 mM EDTA, 0.1 mM EGTA, 0.1% NP-40, and freshly added 1 mM DTT, 1 mM PMSF, 4 μg/ml aprotinin, 2 μg/ml pepstatin) and vortexed 15 min at 4°C. Finally, samples were centrifuged at 20,000 × *g* for 20 min at 4°C and the supernatant transferred in a new tube. This final fraction contains the nuclear extract.

### Immunofluorescence

HVEC were grown on 35 mm glass coverslips until 60–80% confluency and incubated at 37°C with equal amount of serum EVs [healthy patient (c), MGUS, and MM EVs] or only with starvation medium for 4 h.

#### Cellular staining

Cells were washed with PBS 1×, fixed with 3% paraformaldehyde for 15 min, washed with NH_4_Cl for 15 min, and permeabilized with 0.3% saponin in PBS three times for 10 min. Primary antibodies were incubated for 1 h and washed three times for 10 min with 0.3% saponin in PBS 1×. Secondary antibodies were incubated for 45 min and washed as described above. Coverslips were mounted using an anti-fade mounting medium (ProLongGold-Invitrogen) on a glass slide. Fluorescent microscopy was performed on a ZEISS Axiovert 100 microscope using the 63× Zeiss oil immersion objective. Images were processed with the use of Image pro-plus 4.5.1.

#### Nuclear staining

Cells were washed with PBS 1×, fixed with 4% paraformaldehyde for 10 min, permeabilized with 0.2% Triton X-100, 2 mg/ml BSA, 1 mM NaN_3_ in PBS on ice three times for 10 min. Then cells were incubated with blocking solution (0.02% Triton X-100, 3% BSA, 1 mM NaN_3_ in PBS). Primary antibodies were incubated for 30 min in blocking solution and washed three times for 10 min with wash buffer (PBS, 0.02% Triton X-100, 1.5% BSA, 1 mM NaN_3_). Secondary antibodies were incubated for 45 min and washed as described above.

### EVs uptake

To evaluate cells internalization rate of MGUS and MM EVs, HVEC, and H9C2 (40,000 cell/dish of 35 mm) cells were incubated 4 h with PKH67 (Green Fluorescent Cell Linker Kit-Sigma) labeled EVs (200 μg of proteins) at 37°C. As negative controls cells were incubated with PKH67 centrifuged 2 h at 100,000 × *g* without EVs to exclude the presence of PKH67 aggregates. Furthermore, to block endocytosis cells were pre-incubated for 30 min at 4°C or with nocodazole 20 μM (Sigma) at 37°C. After these pre-incubations, PKH67-labeled EVs (200 μg) were added and incubated 4 h, respectively, at 4°C or in the presence of the drug (18). Intracellular uptake was tested by immunofluorescence.

To investigate the EVs uptake mediators, before cell treatment, EVs were incubated with 10 μg of Abs (sheep anti-FLCs or mouse anti-CD63) for 2 h at 4°C and afterwards EVs were ultra-centrifuged to separate unbound Abs. In alternative, serum EVs were pre-treated or not with heparin (Sigma) at a final concentration of 100 ng/ml for 30 min at 4°C. Intracellular uptake was tested by immunofluorescence and/or flow-cytometry.

### Proliferation assay

Eight thousand cells/well (12 multi-well plate) were cultured in RPMI serum-free and control (*n* = 3), MGUS (*n* = 3), MM (*n* = 6) derived EVs (50 μg proteins) were added for 24, 48, and 72 h, respectively. Cell proliferation rates were assessed using crystal violet absorbance. The absorbance values were measured at 540 nm. We calculated the growth factor index with the “doubling time online calculator.” The cell doubling time index determines the dynamics of the cell culture development as average time taken for a cell to complete the cell cycle within several days (24, 48, 72 h). Average of three independent experiments is shown. *T*-test *p-*values were also calculated.

### Antibodies and immunoblotting

The following antibodies were used in our experiments: mouse anti-Hsp 70 (Enzo Life Science), rabbit anti-NfκB (Santa Cruz), rabbit anti c-src (Santa Cruz), mouse anti-CD63 (Millipore), mouse anti-Tsg 101 (Abcam), mouse anti-α-tubulin (Millipore: Mab1637), mouse anti-Annexin V (Santa Cruz), mouse anti-Lamin A/C (Novus), sheep anti-λ FLC and sheep anti-k FLC antibodies (Bethyl Laboratories, USA and the Binding Site, UK), and goat anti-Histone H3 (Santa Cruz).

Extracellular vesicles from patients’ serum were obtained as described before. The supernatants were normalized for protein concentration (Bradford Assay) when possible (in alternative equal volumes of each sample were loaded on a acrylamide–bisacrylamide gel), boiled in reducing SDS sample buffer (80 mM Tris, pH 6.8, 2% SDS, 7.5% glycerol, 0.01% bromophenol blue) supplemented with 2% 2-mercaptoethanol (Sigma) for 5 min at 95°C and separated by SDS-PAGE on a acrylamide/bisacrylamide (10 or 12.5%) gel and analyzed by WB.

To visualize the FLC signal, we performed electrophoresis under native conditions: samples were re-suspended in non-reducing sample buffer (50 mM Tris, 2% SDS, 10% glycerol, 0.01% bromophenol blue pH 6.8) and boiled for 5 min at 95°C.

Afterwards, samples were run in a 12.5% acrylamide–bisacrylamide SDS 0.4% gel, transferred for 1 h onto a PVDF membrane and blocked overnight with 5% fat-free milk, 0.05% Tween-20 in PBS. The PVDF membrane was incubated with the antibodies described above for 2 h in PBS Tween 0.05 + 1% fat-free milk. The membranes were washed 3× for 10 min with PBS Tween 0.05% and incubated for 1 h with one of the following secondary antibodies: rabbit anti-mouse and goat anti-rabbit (Zymed), rabbit anti goat, and donkey anti-sheep (Jackson Immuno Research). Blots were detected using Luminata Classic HRP western substrate (Millipore). Images were acquired using a G:Box Chemi XT Imaging system (Syngene, UK). For densitometric analysis, we took advantage of the Gene Tools (Syngene, UK) software to compare the protein quantification of monoclonal bands.

### Statistical analysis

Significant differences among MGUS datasets and other samples [healthy patient (c) and MM] were determined with Student’s *t*-test (Graph Pad). *p*-Values of <0.05 were considered statistically significant with *p < 0.05, **p < 0.01, and ***p < 0.001. Values were shown as mean ± SEM (Standard error of the mean) of at least three experiments.

Significant differences among different treatments on EVs or cells were determined with Student’s *t*-test (Graph Pad). *p < 0.05, **p < 0.01, and ***p < 0.001.

## Results

### MGUS and MM serum EVs characterization

**Figure 1:**
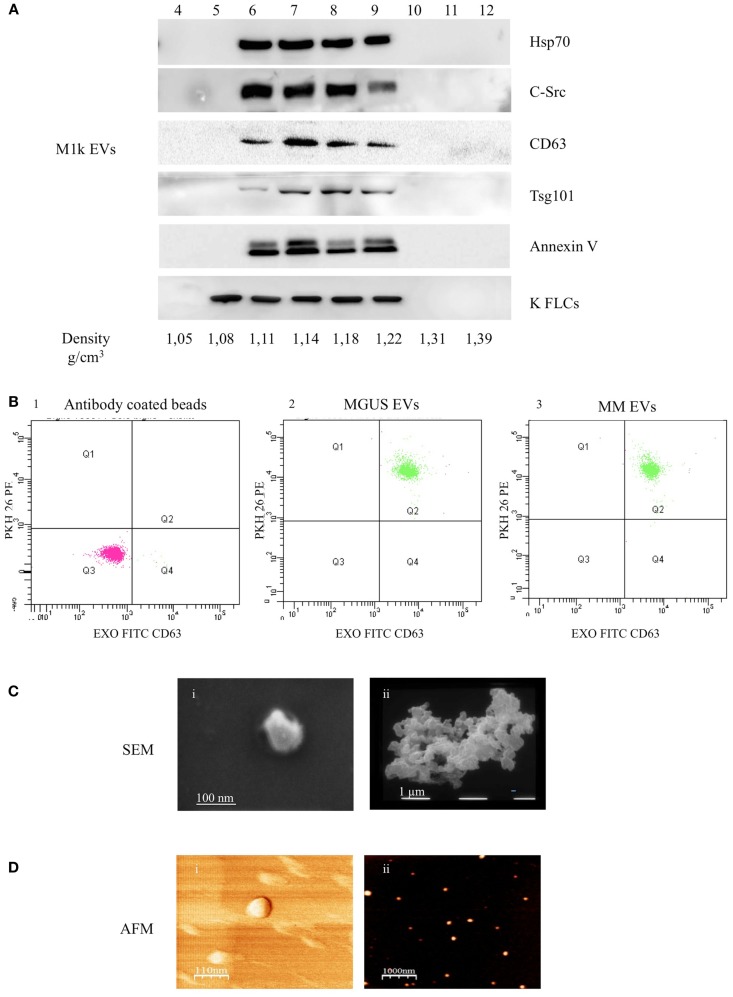
**Serum EVs characterization**. **(A)** Ultra-centrifuged EVs were loaded on the top of a 15–60% discontinuous sucrose gradient. Twelve fractions of equal volume were collected from the top (low density, fraction 1) to the bottom (high density, fraction 12). Samples were electrophoresed and analyzed by WB using anti-HSP70, anti-c-src, anti-CD63, anti-Tsg101, anti-Annexin V, and anti-FLCs antibodies. The EVs probed markers were identified in fractions 6–9 corresponding to the buoyant density of 1.11–1.22 g/cm^3^. **(B)** EVs were purified with CD63 Exo-Flow FACS magnetic beads. CD63 coated EVs were then incubated with PKH26. The data show PKH26 (PE) versus CD63 (FITC) intensity. The first panel (1) depicts antibody coated beads, the second (2) MGUS EVs, and the third (3) MM EVs. **(C)** Scanning electron microscopy (SEM) imaging of the serum EVs: (i) 80,000×, scale bar 100 nm; (ii) 20,000×, scale bar 1 μm. **(D)** Atomic force microscopy (AFM) image of serum EVs: (i) phase, scale bar 110 nm and (ii) topography, scale bar 1 μm.

We purified serum EVs populations from three control subjects (c1–3), three MGUS subjects (MGUS 1–3), and six MM patients (MM 1–6) (Table 1). We decided to use serum instead of plasma for a first line purification because serum does not contain high amounts of coagulation factors that could bind and aggregate EVs. It is well known that EVs from human blood are of high interest because of diagnostic and therapeutic purposes. Commercially available kits that allow “easy isolation procedures” are increasingly developed. Such approaches should be taken cautiously because they often fail to distinguish between differently sized EVs and membrane-free macromolecular aggregates (19). We used a step gradient protocol without filters and characterized the isolated material with AFM, SEM, and flow-cytometry. As shown in Figure 1A, we obtained four gradient fractions (from six to nine) containing some typical exosomal markers and pooled them for subsequent assays and analysis. FACS analysis was performed on PKH26 and CD63 (FITC) labeled EVs and coated with Exo-Flow FACS magnetic beads. MGUS and MM patients presented the same profile in the CD63 expression level (Figure 1B). To better characterize the composition of serum EVs, we performed morphological analyses using SEM. Figure 1C shows the presence of vesicles with diameters from 60 to 100 nm in MM serum samples. The pelleted serum derived vesicles were observed as single vesicles (Figure 1Ci) or vesicles clusters (Figure 1Cii) as previously described for exosomes isolated from different biological fluids (20). More clearly, the punctate pattern in AFM, which was performed on native vesicles, indicates that the EVs were pure and free of aggregates contaminants (Figures 1Di,ii).

**Table 1:** **Description of the patients involved in the study**.

Patients	Disease	k FLC (mg/l)	λ FLC (mg/l)	Ratio	S-IF	U-IF	IgA mg/dl	IgG mg/dl	IgM mg/dl	Creatinine (mg/dl)
c 1	Healthy	18.4	13.5	1.36	neg.	neg.	159	1560	137	0.65
c 2	Healthy	20.1	8.9	2.26	neg.	neg.	96	1130	129	0.51
c 3	Healthy	7.4	17.5	0.42	neg.	neg.	298	1120	122	0.49
MGUS 1	MGUS	1.69	608	0.00	IgG/λ	λ	20.1	2010	28.7	0.6
MGUS 2	MGUS	9.36	284	0.03	IgG/λ	neg.	28.7	2220	15.2	0.9
MGUS 3	MGUS	12.9	105	0.12	λ	neg.	809	845	66.4	0.64
MM 1	Multiple myeloma	1210	22	55.00	IgG/k	k	85.3	729	22.4	0.85
MM 2	Multiple myeloma	1550	4.85	319.59	IgG/k	k	26	978	23.4	2
MM 3	Multiple myeloma	1360	6.15	221.14	IgG/k	k	46.1	519	17.7	0.8
MM 4	Multiple myeloma	26.6	864	0.03	λ	λ	22.6	581	23.7	1.5
MM 5	Multiple myeloma	73.9	1130	0.07	λ	λ	174	1150	142	2.6
MM 6	Multiple myeloma	2.45	4450	0.00	λ	λ	204	727	22.8	0.9

*S-IF, serum immunofixation; U-IF, urine immunofixation; neg., negative*.

### Serum derived MM and MGUS EVs induce different endothelial and myocardial proliferation and internalization rates

**Figure 2:**
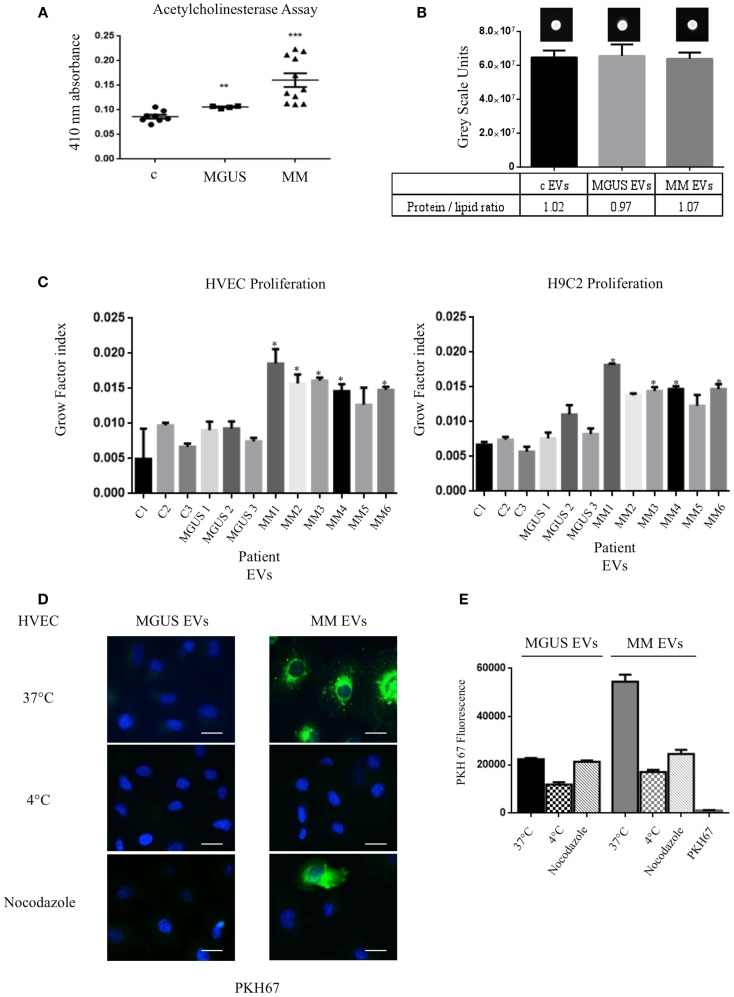
**Internalized MM EVs increase HVEC and H9C2 proliferation**. **(A)** Evaluation of the acetylcholinesterase activity in 50 μg of controls (c, 8 different patients), MGUS (4 patients), and MM (10 patients) crude EVs samples. Significant differences were determined with Student’s *t*-test: *p < 0.05, **p < 0.01, ***p < 0.001. Values were shown as mean SEM of at least three experiments. **(B)** Fluorescent (PKH67-labeled) EVs (200 μg of proteins) from controls, MGUS, and MM patients were re-suspended with loading buffer [36% TAE (Tris–acetate–EDTA), 14% H_2_O, 50% glycerol] to a final volume of 20 μl. Ten microliters were spotted on a nitrocellulose membrane and dots fluorescent signal were acquired using a G:Box Chemi XT Imaging system (Syngene). Signals were quantified with the program Gene Tools. Protein/lipid ratio was quantified for all samples. Mean values ± SEM of at least four different experiments. **(C)** HVEC and H9C2 cells in serum-free medium were incubated with 50 μg of controls (c1–3, 3 different patient), MGUS (1–3), and MM (MM 1–6) EVs for 24, 48, and 72 h and cell proliferation rate (growth factor) was assessed using crystal violet. Mean values ± SEM for three independent experiments are shown. *p < 0.05, **p < 0.01, ***p < 0.001 **(D)** HVECs were incubated with PKH67-labeled MGUS and MM EVs (200 μg of proteins) for 4 h at 37°C; as negative controls cells were pre-incubated for 30 min at 4°C or with nocodazole 20 μM at 37°C followed by 4 h of exposure to EVs at 4°C or in the presence of nocodazole. Cells were then washed with PBS 1× and fixed with 3% PFA, permeabilized with 0.3% saponin, and stained with DAPI. Scale bar 5 μm. Coverslips were mounted using an anti-fade mounting medium (ProLong Gold-Invitrogen) on a glass slide. Fluorescent microscopy was performed on a ZEISS Axiovert 100 fluorescent microscope using the 63× Zeiss oil immersion objective. Single sections are shown for each condition. Images were processed with the use of Image pro-plus 4.5.1. **(E)** PKH67 fluorescence intensity measurement of internalized MGUS and MM EVs before and after endocytosis blockage at 4°C, with nocodazole 20 μM. Internalization rate of PKH67 centrifuged 2 h at 100,000 × *g* without EVs. (100 Cells each experimental point). Images were processed with the use of Image pro-plus 4.5.1. Mean value and SEM of three independent duplicate experiments are given.

In order to verify the amount of EVs in control (c), MGUS, and MM patients, we evaluated the acetylcholinesterase activity in EVs purified from 1 ml of serum after serial centrifugation steps (Figure 2A). MM patients showed the highest enzymatic activity indicating a higher EVs amount than MGUS and c. We decided to normalize the EVs protein concentration on lipid content to load the same EVs amount on cells. EVs from 1 ml serum from control (three patients), MGUS (three patients), and MM (six patients) were pelleted after serial centrifugation steps. EVs protein concentration was determined. Samples were normalized for their protein content (200 μg) of pelletted proteins, EVs lipids were labeled with PKH67 and spotted on a nitrocellulose membrane (Figure 2B). Dot fluorescence intensity was acquired using a G:Box Chemi XT Imaging system (Syngene) and signals were quantified with the Gene Tools program. Samples protein content was normalized on the lipid fluorescent signal and ratios were almost identical for the different EVs preparations (protein/lipid ratio controls 1.02, MGUS 0.97, MM 1.07, Figure 2B). Consequently, 50 μg of EVs obtained from control, MGUS, and MM patients were incubated 24, 48, and 72 h respectively, at 37°C, on endothelial and myocardial monolayers. Cell proliferation rates were assessed using crystal violet absorbance. The growth factor index was calculated with the “doubling time online calculator” and represented as scale bars (Figure 2C). Statistical analysis showed a significant difference between cell monolayers incubated in the presence of different EVs. Particularly, MM EVs clearly enhanced the proliferation rates. These data are of interest for further investigation on patients with amyloidosis because heart failure is the major negative prognostic factor in such patients (21).

To investigate if the EVs inducible proliferation is uptake dependent, we incubated endothelial monolayers with PKH67 labeled EVs (200 μg) for 4 h at 37°C. Images of washed and fixed cells clearly show that only few amounts of MGUS EVs were internalized in endothelial cells whereas active MM EVs uptake is evident (Figures 2D,E). This effect is endocytosis-mediated, MM EVs show an uptake decrease of 69 and 53%, respectively, when cells are incubated at 4°C or with the endocytosis inhibitor nocodazole. We also tested EVs uptake specificity, incubating cells with PKH67 without EVs and, in this condition, the fluorescent label is not internalized (Figure 2E). Same experiments were performed with H9C2 cells and gave similar results (data not shown).

### FLCs and GAGs mediate MM EVs intracellular uptake

**Figure 3:**
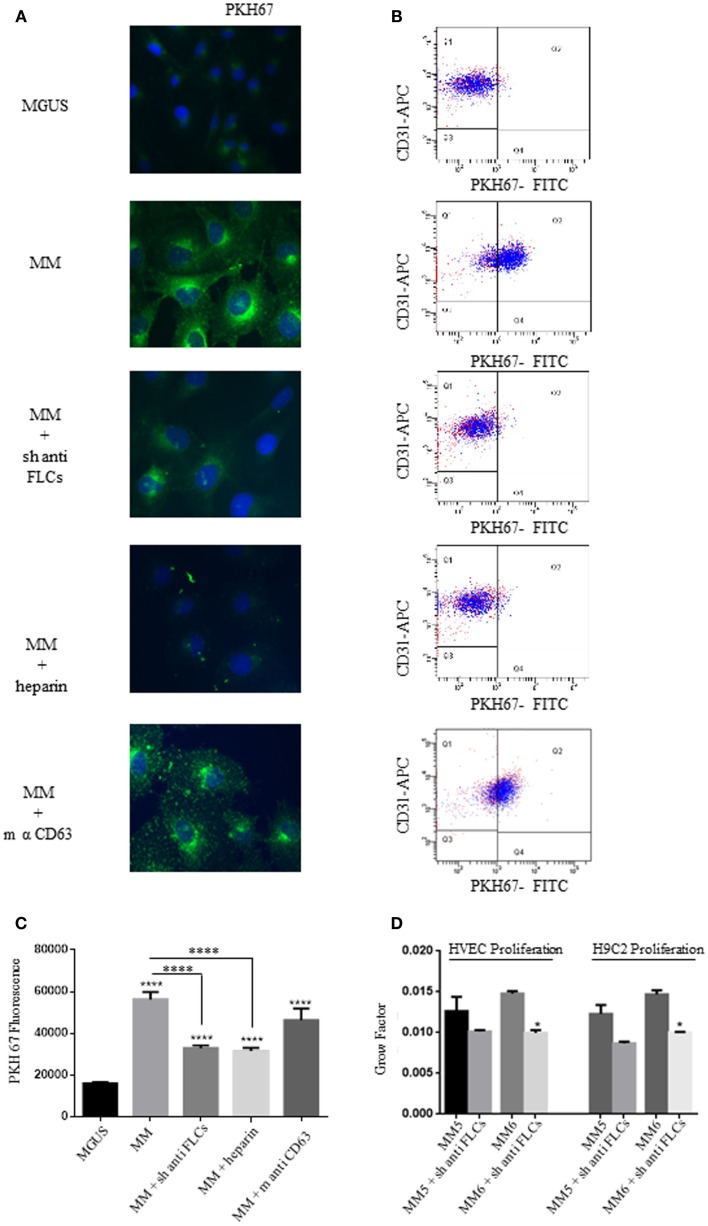
**Free light chains and GAGs mediate MM EVs intracellular uptake**. **(A)** Fluorescent microscopy analysis of HVECs incubated with MGUS and MM PKH67-labeled EVs (200 μg of proteins) for 4 h at 37°C. Before cell treatment, MM EVs were incubated with sheep anti-FLCs (MM + sh anti FLC), mouse anti-CD63 (MM + sh anti-CD63) antibody or with heparin 100 ng/ml (MM + heparin). **(B)** Quantitative data from similar experiments analyzed by flow cytometry. HVECs were labeled with CD31-APC (endothelial marker) and EVs were labeled with PKH67-FITC. **(C)** PKH67 fluorescence intensity measurement of internalized MM EVs incubated with sheep anti-FLCs, mouse anti-CD63 antibody or with heparin 100 ng/ml (MM + heparin) (100 cells each experimental point) using ImageJ program. Significant differences were determined with Student’s *t*-test. Values were shown as mean values ± SEM of at least three experiments. *p < 0.05, **p < 0.01, ***p < 0.001. **(D)** HVEC and H9C2 cells in serum-free medium were incubated for 24, 48, and 72 h with 50 μg of MM 5 and MM 6 EVs pre-treated or not with a polyclonal anti-FLCs antibody. Cell proliferation rate (growth factor) was assessed using crystal violet. Mean values ± SEM for three independent experiments are shown. *p < 0.05, **p < 0.01, ***p < 0.001.

The long-distance action of EVs is probably related to specific docking signals in recipient cells. Autocrine models have also been studied in the modulation of tumor microenvironment. To test if the different intracellular uptake of MGUS and MM EVs and the diverse proliferation rates are directly linked, we sought some targeting molecules to be blocked in EVs docking and subsequent internalization. The presence of FLCs on the external surface of MM EVs (5) suggested us to test the cellular uptake after incubation of EVs with polyclonal antibodies against FLCs. Before cell treatment EVs were incubated with the specific Abs (anti-FLCs or anti-CD63) for 2 h and afterwards EVs were ultra-centrifuged to separate unbound Abs. The pelleted EVs were added in the serum-free cell culture medium and intracellular uptake tested by immunofluorescence (Figure 3A) and flow-cytometry (Figure 3B). As shown in Figure 3A, the internalized EVs are clearly reduced after anti-FLCs treatment. Quantification of positive stained cells was significantly altered (*p* < 0.001) in comparison with MM EVs treated cells (Figure 3C). To confirm the confocal microscopy evidences we trypsinized cell monolayers after EVs intake and evaluated the PKH67 intensity versus positive CD31 staining by flow cytometry. Our data show that only few amounts of FLCs masked EVs are visible in the dot plot analysis of endothelial cells (Figure 3B). The same results were obtained with heparin pre-treated EVs but not with an anti-CD63 antibodies (Figures 3A–C). To investigate the active internalization of EVs and subsequent release of their content as a trigger factor of increased proliferation rates, we performed the proliferation assay with or without FLCs masked MM EVs (Figure 3D) on endothelial and myocardial cells and we could observe that masked MM EVs do not induce the same proliferation rate as MM EVs do.

### MM EVs induced c-src re-distribution and NfκB nuclear translocation are intracellular uptake dependent

Endocytosis of monoclonal FLCs into the kidney proximal tubular epithelium promotes a c-src and NfκB dependent generation of an intrarenal pro-inflammatory, profibrotic process (22). This pathway promotes the pro-inflammatory production of other cytokines, such as IL-6 enhancing kidney damage in MM (23–26). The presence of EVs containing FLCs in urine samples has also been published (27) suggesting that EVs are probably involved in different FLCs processing and subsequent inflammation pathways. To study the potential EVs induced pro-inflammatory response of endothelial cells, we treated HVECs with MGUS and MM EVs for 4 h at 37°C. Afterwards, we performed an immunofluorescence assay (see Materials and Methods) to visualize c-src and NfκB intracellular distribution.

**Figure 4:**
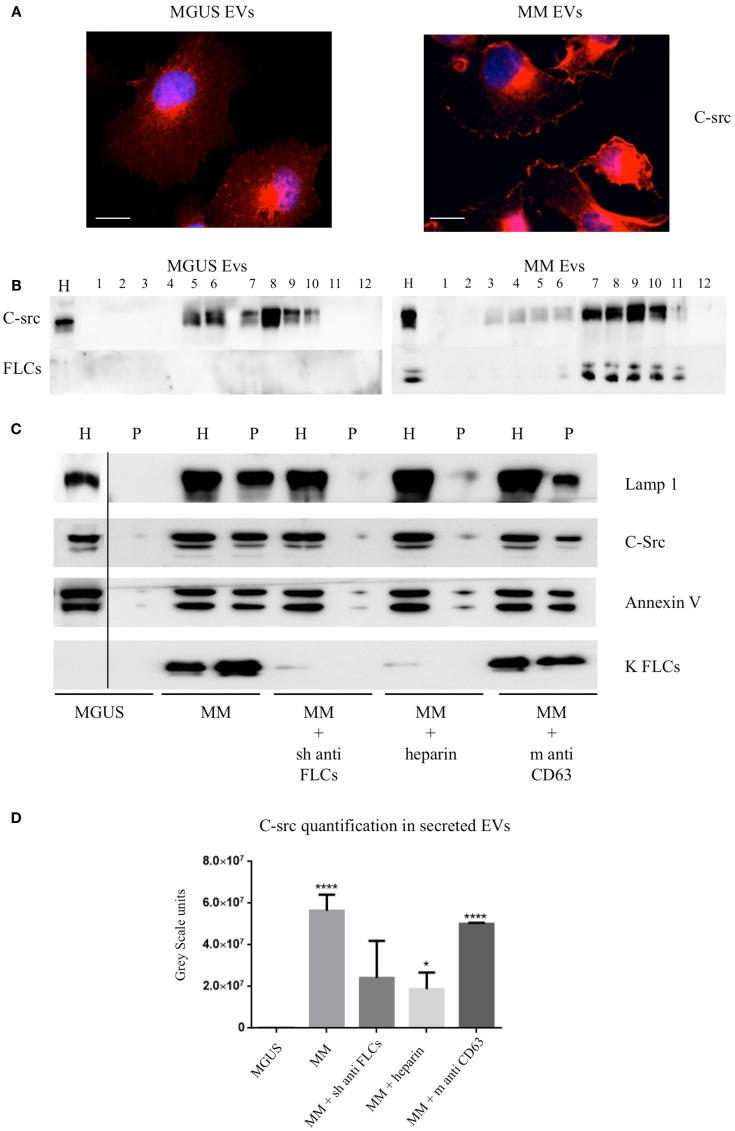
**C-src re-distribution in HVECs after EVs exposure**. **(A)** HVECs were incubated with 200 μg of MGUS and MM serum EVs for 4 h at 37°C. Cells were washed with PBS 1× and fixed with 3% PFA, permeabilized with 0.3% saponin, and stained with the c-src antibody, followed by Alexa 555-conjugated anti-rabbit immunoglobulin (Ig) and DAPI. Scale bars, 5 μm. Coverslips were mounted using an anti-fade mounting medium (ProLong Gold-Invitrogen) on a glass slide. Fluorescent microscopy was performed on a ZEISS Axiovert 100 fluorescent microscope using the 63× Zeiss oil immersion objective. Single sections are shown for each condition. Images were processed with the use of Image pro-plus 4.5.1. **(B)** HVECs were treated as described above and lysates were loaded on the top of a 15–60% discontinuous sucrose gradient. Twelve fractions of equal volume were collected from the top (low density, fraction 1) to the bottom (high density, fraction 12). Samples were electrophoresed and analyzed by WB using anti-c-src and anti-FLCs antibody. **(C)** 10 × 10^6^ HVECs were incubated for 4 h at 37°C with 200 μg MGUS and MM EVs pre-treated or not with a polyclonal anti-FLCs antibody (MM + sheep anti-FLCs), mouse anti-CD63 antibody (MM + m anti-CD63) or with 10 μg/ml heparin for 2 h at 4°C (MM + heparin). After incubation, cells were then washed with PBS 1× and treated with trypsin as described (5) and left in fresh starvation medium for 16 h. Starvation medium was harvested, centrifuged at 800 × *g* for 30 min, 16,000 × *g* for 45 min, and finally, ultra-centrifuged at 100,000 × *g* for 2 h (see EVs Purification and Fractionation, Materials and Methods). WB analysis of cell extracts (homogenate, H, 30 μg) and pellets (p, 25 μl) were performed with anti-FLCs antibodies and with different vesicles markers (Lamp1, c-src, annexin V, and FLC). **(D)** c-src quantification in pellets after different treatments. Significant differences among the samples were determined with Student’s *t*-test: *p < 0.05, **p < 0.01, ***p < 0.001. Values were shown as mean values ± SEM of at least three experiments.

The intracellular c-src distribution was clearly altered after MM EVs exposure instead of MGUS EVs. Immunofluorescence analysis showed prevalent plasma membrane localization (Figure 4A). Sucrose gradient fractionation of the cell homogenates indicates a c-src re-distribution from lower to higher density fractions (Figure 4B) confirming the immunofluorescence data.

We previously published that (5) MM EVs are able to induce cellular *ex novo* secretion of c-src positive EVs, thus, we tested if anti-FLCs antibodies and heparin were both able to block the c-src decorated EVs production. We observed a striking reduction of c-src positive EVs generation and we could not see the same effect using a different target such as CD63 (Figures 4C,D).

**Figure 5:**
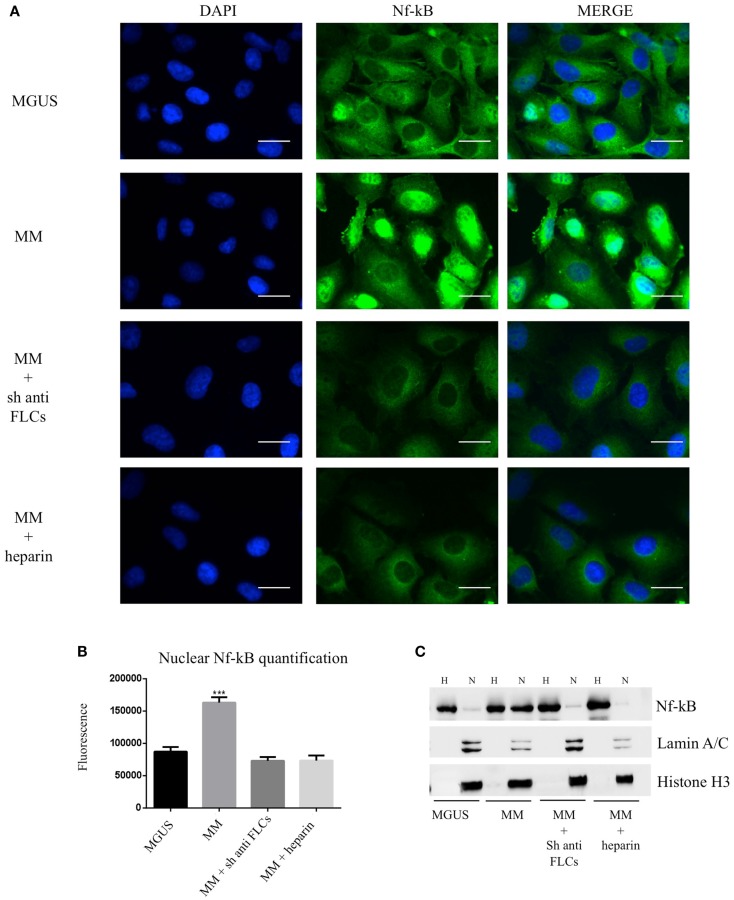
**Visualization of NfκB nuclear traslocation (A) HVECs were treated with MGUS or MM serum EVs (200 μg of proteins) for 4 h at 37°C**. Before cell treatment, MM EVs were incubated with anti-FLCs antibody (MM + sh anti FLC) or with 100 ng/ml of heparin (MM + heparin). After incubation, cells were washed with PBS 1× and fixed with 4% PFA, permeabilized with 0.2% Triton X-100, 2 mg/ml BSA, 1 mM NaN_3_ in PBS, and stained with anti-NfκB antibody, followed by Alexa 488-conjugated anti-rabbit immunoglobulin (Ig) and DAPI. Scale bars, 5 μm. Coverslips were mounted using an anti-fade mounting medium (ProLong Gold-Invitrogen) on a glass slide. Fluorescent microscopy was performed on a ZEISS Axiovert 100 fluorescent microscope using the 63× Zeiss oil immersion objective. Single sections are shown for each condition. Images were processed with the use of Image pro-plus 4.5.1. **(B)** Nuclear NfκB fluorescence intensity was measured after different treatments (100 cells each experimental point) using ImageJ program. Significant differences were determined with Student’s *t*-test: *p < 0.05, **p < 0.01, ***p < 0.001. Values were shown as mean values ± SEM of at least three experiments. **(C)** WB analysis of cell extracts (homogenate, H, 25 μg) and nuclear extract (N, 50 μg) were performed with anti-NfκB antibody and with different nuclear markers (Lamin A/C and Histone H3).

**Figure 6:**
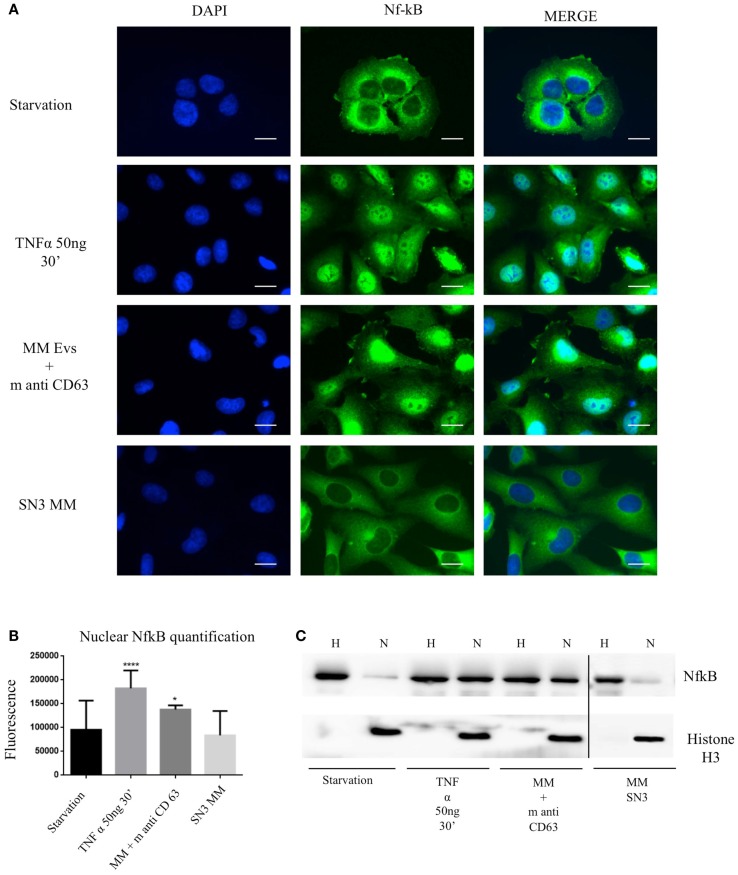
**Extracellular vesicles induce NfκB nuclear traslocation**. **(A)** HVECs were treated with serum-free medium for 4 h (starvation), 10 ng/ml TNF-α for 30 min, with MM EVs pre-incubated with anti-CD63 antibody (MM + m anti-CD63) or with MM serum after serial ultracentrifugation steps (SN3 MM). Cells were fixed with 4% PFA, permeabilized with 0.2% Triton X-100, 2 mg/ml BSA, 1 mM NaN_3_ in PBS, and stained with anti-NfκB antibody, followed by Alexa 488-conjugated anti-rabbit immunoglobulin (Ig) and DAPI. Scale bars, 5 μm. Coverslips were mounted using an anti-fade mounting medium (ProLong Gold-Invitrogen) on a glass slide. Fluorescent microscopy was performed on a ZEISS Axiovert 100 fluorescent microscope using the 63× Zeiss oil immersion objective. Single sections are shown for each condition. Images were processed with the use of Image pro-plus 4.5.1. **(B)** Nuclear NfκB fluorescence intensity was measured after different treatments (100 cells each experimental point) using ImageJ program. Significant differences were determined with Student’s *t*-test: *p < 0.05, **p < 0.01, ***p < 0.001. Values were shown as mean values ± SEM of at least 3 experiments. **(C)** WB analysis of cell extracts (homogenate, H, 25 μg) and nuclear extract (N, 50 μg) were performed with anti-NfκB antibody and with anti-Histone 3 antibody.

To test the effect of MM EVs on NfκB, we treated HVECs with MGUS and MM EVs for 4 h at 37°C (Figure 5A). Only HVEC incubated with MM EVs showed a strong NfκB nuclear traslocation. The effect was completely abolished with MM EVs pre-treated with anti-FLCs antibodies and heparin. We quantified the nuclear NfκB fluorescent signal (Figure 5B) and we could observe that cells incubated with MM EVs showed a significant increase in comparison with other treatments. These data were also strongly confirmed by WB analysis of nuclear extracts (Figure 5C). To test the efficacy of the NfκB nuclear traslocation, we also compared our data with a TNFα pro-inflammatory treatment and with MM EVs pre-coated with the CD63 antibody: as expected we obtained a significant NfκB nuclear traslocation compared to control cell (starvation) both in immunofluorescence and WB analysis (Figures 6A–C). To confirm that NfκB nuclear traslocation is specifically induced by MM EVs, we incubated HVECs with MM serum after the serial ultracentrifugation steps (SN3). Figures 6A–C show that this treatment do not significantly alter NfκB intracellular distribution in comparison with control cells.

## Discussion

In the present study, we found that the intracellular uptake of serum MM EVs is enhanced in comparison to MGUS EVs. Our data show that MM EVs internalization is FLCs and GAGs mediated and we could demonstrate that MM EVs induce the NfκB nuclear translocation and the c-src kinase containing EVs generation. Blocking FLCs with anti-FLCs antibodies or masking the GAGs recognition with heparin altered the EVs intracellular uptake, the NfκB nuclear translocation, and the c-src positive EVs secretion.

Extracellular vesicles involvement in hematologic malignancies such as MM is an emerging multifaceted tool for both diagnosis and therapy (28, 29). The evidences that cancer cells can modulate metastasis generation by increased and specific EVs secretion led to the investigation of the interaction between BM–MSC and MM cells. A mechanism that modulates MM tumor growth was identified in the exosomal transfer from BM–MSCs to clonal plasma cells: MM BM–MSC-derived exosomes promoted MM tumor growth whereas normal BM–MSC exosomes inhibited the growth of MM cells (3).

We previously found that circulating EVs are significantly increased in MM patients in comparison to MGUS subjects and we identified the c-src kinase as a specific MM EVs marker (5).

In the present work, we investigated the effect of circulating MM EVs on endothelial and myocardial cells: to this aim, we performed differential ultracentrifugation protocols for serum EVs purification. To be sure that small protein aggregates were removed we checked our EVs preparations with morphological assays, combining SEM and AFM.

Functional activities of EVs can be executed at different levels such as signaling through cell-surface receptors, transfer of signaling proteins, intracellular release of miRNA, mRNA, and DNA, or distribution of catalytic activities (30). Upon loading and release EVs are targeted to recipient cells in a not well understood fashion. Specific receptors or docking systems may contribute to the pathophysiology of tumor progression. Thus, we tested first of all the internalization rate of purified MGUS and MM serum EVs in endothelial and myocardial cells. We strikingly found that MM EVs are internalized while MGUS EVs are not, leading to the hypothesis that MM EVs can behave as signalosomes in the blood stream.

Heparan sulfate, proteoglycans have been shown to function as internalizing receptors of cancer cell-derived EVs (9) and are further involved in immunoglobulin FLCs amyloidogenesis (10). Because FLCs are actively internalized (31–33), we decided to test their involvement in EVs uptake together with GAGs and we found that both are responsible in efficient EVs uptake.

Nuclear factor kappa B transcription factors play a key role in the survival and proliferation of many kinds of B-cell tumors, including MM. It was shown that NfκB activation in MM tumors results mainly from extrinsic signaling that stimulate receptors on normal plasma cells as well as on pre-malignant MGUS and MM tumors (34). The NfκB signaling has been rationalized in two independent signaling pathways, the canonical and not canonical that both induce NfκB nuclear translocation and genes transcription. The Src-family kinases are also involved in such signaling cascades ultimately leading to cell survival and proliferation (35). Interestingly, NfκB is constitutively active in primary myeloma cells, and its blockade leads to apoptotic cell death (36).

In our investigation on the pathological effect of MM EVs, we found that they are capable to induce higher proliferation rates and to translocate the NfκB into the nucleus. Blocking MM EVs uptake with polyclonal antibodies directed against FLCs or by EVs heparin coating clearly abolished the effect. Similar results were obtained after treating cells with the endocytosis inhibitor nocodazole (data not shown). It will be extremely interesting to test the relevance of EVs induced NfκB nuclear translocation in monitoring MGUS versus MM switching on a larger patient cohort. Further work is in progress to unravel the major EVs component involved in this signaling process. Our evidences that MM serum derived EVs contain the c-src kinase and generate new c-src positive vesicles in cultured cells suggest a potential “signalosomal” function of EVs in MM. The antitumor action of chemically modified heparins has been shown to inhibit myeloma growth and angiogenesis via disruption of the heparanase/syndecan-1 axis (37), and our data strongly confirm the usefulness of this therapeutic approach. The evidence that we saw on FLCs involvement in EVs uptake open a new door for combined therapeutic approaches, particularly in patients with FLCs induced kidney failure or amyloidosis.

Multiple myeloma is one of the most frequent hematological malignancies and combined therapeutic approaches are needed to ameliorate the life expectancy of MM patients.

Our study shows for the first time that FLCs are involved in EVs intracellular uptake and confirm that GAGs are also involved in tumor derived EVs processing. The NfκB nuclear translocation after MM EVs exposure demonstrate the transactivating role of EVs in cancer and our discovery that anti-FLCs antibodies and heparin are able to block this pathway open new insights in EVs cellular biology, MM therapeutic and diagnostic approaches.

## Author Contributions

Doris Ricotta, Giuseppe Di Noto conceived and designed the experiments. Giuseppe Di Noto, Marco Chiarini, Lucia Paolini, Elena Laura Mazzoldi, and Viviana Giustini performed the experiments. Giuseppe Di Noto, Lucia Paolini, and Annalisa Radeghieri were involved in samples recruitment. Doris Ricotta, Giuseppe Di Noto, Marco Chiarini, Lucia Paolini, Viviana Giustini, Luigi Caimi, and Annalisa Radeghieri analyzed the data. Doris Ricotta and Giuseppe Di Noto wrote the first draft of the manuscript. All authors reviewed and edited the manuscript and approved the final version of the manuscript.

## Conflict of Interest Statement

The authors declare that the research was conducted in the absence of any commercial or financial relationships that could be construed as a potential conflict of interest.
